# The effect of pressure support on imposed work of breathing during paediatric extubation readiness testing

**DOI:** 10.1186/s13613-019-0549-0

**Published:** 2019-07-02

**Authors:** Jefta van Dijk, Robert G. T. Blokpoel, Alette A. Koopman, Sandra Dijkstra, Johannes G. M. Burgerhof, Martin C. J. Kneyber

**Affiliations:** 1 Division of Paediatric Critical Care Medicine, Department of Paediatrics, Beatrix Children’s Hospital, University Medical Center Groningen, University of Groningen, Internal Postal Code CA 62, P.O. Box 30.001, 9700 RB Groningen, The Netherlands; 2 Department of Epidemiology, University Medical Center Groningen, University of Groningen, Groningen, The Netherlands; 3 Critical Care, Anaesthesiology, Peri-operative and Emergency Medicine (CAPE), University of Groningen, Groningen, The Netherlands

**Keywords:** Child, Mechanical ventilation, Imposed work of breathing, Extubation readiness test, Pressure support, Paediatric intensive care

## Abstract

**Background:**

Paediatric critical care practitioners often make use of pressure support (PS) to overcome the perceived imposed work of breathing (WOBimp) during an extubation readiness test (ERT). However, no paediatric data are available that shows the necessity of adding of pressure support during such tests. We sought to measure the WOBimp during an ERT with and without added pressure support and to study its clinical correlate. This was a prospective study in spontaneously breathing ventilated children < 18 years undergoing ERT. Using tracheal manometry, WOBimp was calculated by integrating the difference between positive end-expiratory pressure (PEEP) and tracheal pressure (Ptrach) over the measured expiratory tidal volume (VTe) under two paired conditions: continuous positive airway pressure (CPAP) with and without PS. Patients with post-extubation upper airway obstruction were excluded.

**Results:**

A total of 112 patients were studied. Median PS during the ERT was 10 cmH_2_O. WOBimp was significantly higher without PS (median 0.27, IQR 0.20–0.50 J/L) than with added PS (median 0.00, IQR 0.00–0.11 J/L). Although there were statistically significant changes in spontaneous breath rate [32 (23–42) vs. 37 (27–46) breaths/min, *p* < 0.001] and higher ET-CO_2_ [5.90 (5.38–6.65) vs. 6.23 (5.55–6.94) kPa, *p* < 0.001] and expiratory Vt decreased [7.72 (6.66–8.97) vs. 7.08 (5.82–8.08) mL/kg, *p* < 0.001] in the absence of PS, these changes appeared clinically irrelevant since the Comfort B score remained unaffected [12 (10–13) vs. 12 (10–13), *P* = 0.987]. Multivariable analysis showed that changes in WOBimp occurred independent of endotracheal tube size.

**Conclusions:**

Withholding PS during ERT does not lead to clinically relevant increases in WOBimp, irrespective of endotracheal tube size.

**Electronic supplementary material:**

The online version of this article (10.1186/s13613-019-0549-0) contains supplementary material, which is available to authorized users.

## Background

Assessment of extubation readiness in mechanically ventilated children remains challenging despite the relatively low failed extubation rate (2–20%) [1–4]. Patients who failed extubation may experience prolonged intensive care stay and even increased mortality [5]. This signifies the importance of appropriately identifying when the patient is ready for extubation. Extubation readiness testing (ERT) (i.e. a formal trial of spontaneous breathing) is a key component in the process of discontinuing mechanical ventilation (MV). ERTs can be done using continuous positive airway pressure (CPAP) with or without added pressure support (PS) or with a T-piece. To date, no paediatric data support superiority of one type of ERT over the other, although most paediatric critical care practitioners use CPAP with added PS during the ERT [6, 7]. This practice is based on the perceived added resistance of the patient circuit and smaller endotracheal tube (ETT) in young children, leading to increased respiratory workload [8]. Indeed, bench testing showed that the resistance in the smallest ETT is larger when matched for flow compared to larger ETTs, although higher flow rates were tested then the 0.5 mL/kg generated by children [9].

At the same time, the practice of adding PS may also be questioned. A recent meta-analysis of 16 studies examining patient effort during various spontaneous breathing trials (SBT) confirmed that although PS reduced respiratory effort, only using T-piece (or CPAP 0 cmH_2_O) more accurately reflected physiologic conditions after extubation [10]. Observational studies in children showed that SBTs with PS did not lead to increased physiologic WOB compared to those done without PS [11–13]. Furthermore, ERT outcome and post-extubation work of breathing (WOB) were underestimated when PS was added to the SBT in children [14, 15]. More recently, Khemani and colleagues reported similar pre- and post-extubation pressure-rate products (PRP) as proxy for total WOB (WOBtot) when comparing CPAP with added PS versus CPAP alone in 409 mechanically ventilated children [15]. These studies suggest that SBTs should be done without using PS.

WOBtot not only includes the physiologic WOB (WOBphys), but also entails the work a patient has to generate to overcome the resistive properties of the ETT and patient circuit. The energy to overcome this is coined imposed WOB (WOBimp), which is calculated by integrating the difference between positive end-expiratory pressure (PEEP) and tracheal pressure (Ptrach) over the measured tidal volume (VTe). To date, clinicians do not routinely measure Ptrach necessary for calculating WOBimp, making it difficult to determine what causes increased WOB during a SBT (i.e. WOBimp or WOBphys) [16]. In the present study, we measured WOBimp in a heterogeneous group of mechanically ventilated children to test the hypothesis that the increase in WOBimp when a patient is on CPAP alone does not lead to increased patient discomfort and would therefore be clinically irrelevant.

## Methods

### Patients

This study was designed as a prospective, observational study in invasively mechanically ventilated children admitted to the paediatric intensive care unit (PICU) of the Beatrix Children’s Hospital between March 2017 and June 2018 who were identified by the attending physician to be ready for extubation. Our clinical algorithm describes weaning as follows: weaning starts when ventilator pressures and/or mandatory breath rate can be decreased. During this process, patients are assessed daily during morning rounds by the attending physician for extubation readiness (i.e. able to breathe spontaneously when on CPAP/PS with pressure support < 12 cm H_2_O, FiO_2_ < 0.4 and an adequate coughing reflex). Patients were eligible if they have been invasively ventilated for at least 24 h and the attending physician confirmed extubation readiness and extubation was expected within 8 h. For logistical reasons, patients were only studied on weekdays from 7 am to 5 pm if they had been intubated > 24 h prior to the ERT. Patients with depressed respiratory drive inherent to congenital or acquired central nervous system disorders, congenital or acquired injury to the phrenic nerve or diaphragmatic dysfunction, unstable haemodynamics (i.e. increase in vasoactive support or fluid boluses < 6 h before ERT), congenital or acquired neuro- and/or myopathy, continuous muscular paralysis 12 h before the ERT, patients who had a tracheostomy and patients with ETT leakage > 20% were not studied. Importantly, patients with clinically identified post-extubation upper airway obstruction were removed from analysis because we also wanted to explore the relationship between WOBimp and extubation outcome. The institutional review board (IRB) approved the study and waived the need for informed consent.

### Measurement protocol

Patients were intubated with a cuffed ETT (KimVent, Microcuff Endotracheal Tube, Paediatrics, Roswell, USA) and ventilated with the AVEA^®^ ventilator (CareFusion, Yorba Linda, CA, USA). Prior to the ERT, a 3.5 French (Fr) catheter for ETT < 4.5 mm and 5 Fr for ETT ≥ 4.5 mm (Argyle, Covidien, Mansfield, USA) with the tip of the catheter at the distal end of the ETT was inserted. The patient was then switched to CPAP/PS with the level of PS set similar to the added pressure above the level of PEEP during controlled MV, targeting an expiratory Vt of 5–7 mL/kg actual bodyweight (as there was no obesity in the patient cohort). Vt was measured at the Y-piece of the patient circuit using a self-calibrating pneumotachometer (VarFlex™, CareFusion, Yorba Linda, CA, USA). Flow trigger was set between 0.5 and 1.0 L/min. A heat moisture exchanger (Gibeck, Teleflex Medical, Vianen, The Netherlands) was in situ between the patient circuit and the ETT.

After a 5-min stabilisation period, data were recorded during 5 min of steady-state breathing. Subsequently, PS was turned down to zero and, after a 5-min stabilisation period, again data were recorded during a period of 5 min steady-state breathing.

Ventilator recordings were sampled at 100 Hz using the VOXP protocol and a custom-build software program (Polybench, Applied Biosignals, Weener, Germany).

Heart rate (HR), respiratory rate (RR), peripheral saturation (SpO_2_) and fraction inspired oxygen (FiO_2_) were recorded on case record forms at baseline (i.e. after the first 5-min stabilisation period), after 5 min of steady-state breathing on CPAP/PS, and after 5 min of steady-state breathing on CPAP. The Comfort B score was calculated at these same time points to assess patient comfort [17]. Demographic and baseline clinical data were collected to characterise the studied population included gender, age, weight, 24-h paediatric RISK of mortality (PRISM) III score, admission diagnosis and ETT size [18].

Extubation failure was defined as the need for reintubation within 48 h or use of non-invasive ventilation (NIV) post-extubation.

### Data analysis

Ventilator recordings were analysed offline using a custom-build MatLab script (MATLAB 2018a, The Mathworks, Natick, USA). The median (IQR) of respiratory variables including peak inspiratory pressure (PIP), Ptrach, PEEP, mean airway pressure (mPaw), expiratory Vt number of breaths, RR, rapid shallow breathing index (RSBI), end-tidal CO_2_ (ET-CO_2_), peak inspiratory flow rate (PIFR) and WOBimp was calculated for the 5-min recordings after removal of artefacts. Peak inspiratory resistance (cmH_2_O/L/S) was calculated using ETT size (3.0 mm–6.0 mm) and PIFR using formulae used by Khemani et al. [15].

### Statistical analysis

Data were assessed for normality using the Kolmogorov–Smirnov test. Descriptive data were expressed as median (25–75 interquartile range) or percentage (%) of total. For the univariate analysis, data recorded during CPAP/PS were compared with data recorded during CPAP alone using the Wilcoxon signed rank test. Subsequently, multivariate linear regression analysis using backward selection was performed to study the independent contribution of ETT size, VTe, inspiratory time (Tinsp) and PIFR to changes in WOBimp (∆WOBimp) because we presumed these variables to be related to WOBimp. Statistical analysis was performed using SPSS v23 (IBM, Armonk, NY, USA)*. P* values < 0.05 were considered statistically significant.

## Results

**Fig. 1:**
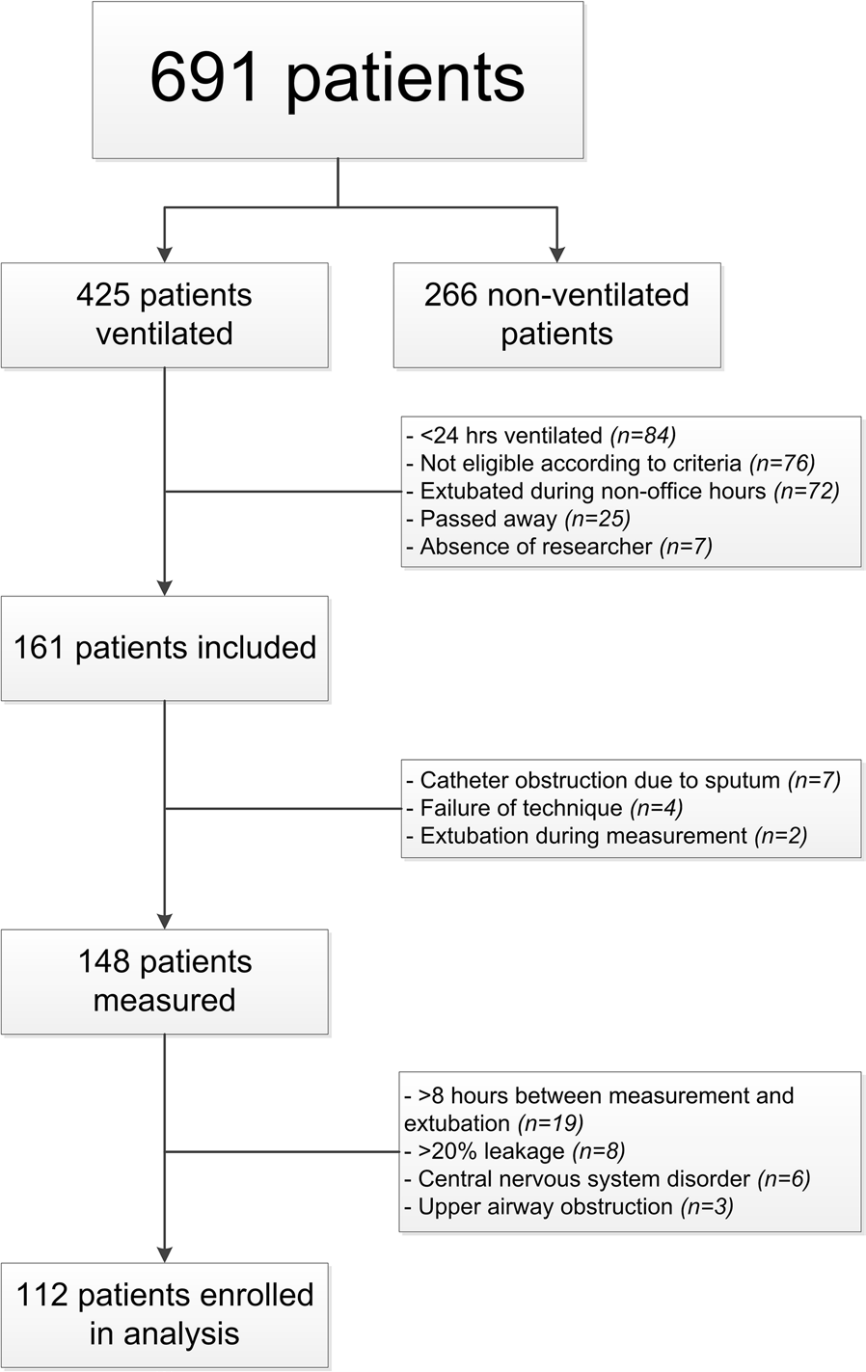
Flow diagram of the cohort

A total of 691 patients were admitted of whom 425 patients were mechanically ventilated. One hundred and sixty-one (37.9%) of these were studied of whom three failed the ERT; ultimately, data of 112 patients were eligible for analysis (Fig. 1). Table 1 summarises the patient characteristics for these 112 patients. Median age was 7.8 months (IQR 2.6–30.6), with almost two-thirds of patients being < 1 year. Nearly half of the patients (43.7%) suffered from an acute respiratory disorder, whereas 37 (33.0%) patients were admitted post-operatively after cardiac surgery. Prior to the ERT, about half (48.2%) of the patients were already weaned using CPAP/PS, whereas 58 patients (46.4%) were ventilated with pressure control (PC) assist/control (A/C) or PC/synchronised intermittent mandatory ventilation (SIMV) with PS (Additional file 1, Table 1). The median PS was 10 (IQR 10–12) cmH_2_O. Median ventilation time for the cohort was 68 (IQR 24–131) h. Nine patients (8%) had failed extubation and were reintubated.

**Table 1:** Characteristics of the cohort

Variable	*N* (%) or median (IQR) *N *= 112
Male	67 (59.8%)
Weight (kg)	7.9 (4.6, 12.9)
Age	
Overall (years)	0.65 (0.22, 2.55)
0–1 month	13 (11.6%)
1–6 months	37 (33.0%)
6–12 months	20 (17.9%)
1–2 years	10 (8.9%)
2–7 years	20 (17.9%)
7–12 years	6 (5.4%)
> 12 years	6 (5.4%)
Admission diagnosis	
Respiratory	49 (43.7%)
Cardiac surgery	37 (33.0%)
Other surgery	17 (15.2%)
Haemodynamically	3 (2.7%)
Neurologic	1 (0.9%)
Other*	5 (4.5%)
Admission characteristics	
Admission time (days)	5.12 (2.24, 7.80)
Ventilation time (days)	2.85 (1.00, 5.47)
PRISM III	3.00 (1.00, 5.00)
PIM II	− 3.77 (− 4.32, − 3.17)

Data are shown as number (% of total) or median (interquartile range)

*Trauma, intoxication, drowning and eating disorder

### Effect of PS on clinical variables and WOBimp

**Fig. 2:**
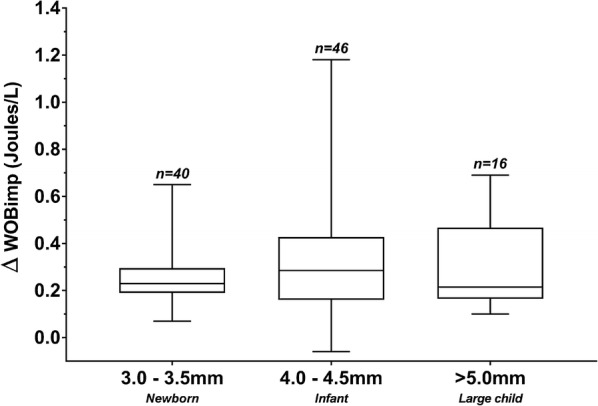
Difference in imposed work of breathing (∆WOBimp) expressed in Joules/L during extubation readiness testing using continuous positive airway pressure (CPAP) with or without added pressure support (PS) stratified by endotracheal tube (ETT) size. Data are shown as median (IQR)

When patients were on CPAP alone compared to CPAP/PS, they had a significantly higher spontaneous breath rate (*p* < 0.001), higher ET-CO_2_ (*p* < 0.001) and significantly lower expiratory Vt (*p* < 0.001) (Table 2). WOBimp was significantly lower when patients were on CPAP/PS [0.00 (0.00–0.11) J/L] compared with CPAP without PS [0.27 (0.20–0.50) J/L]. When stratified by ETT size, the difference in WOBimp between CPAP/PS and CPAP without PS (∆WOBimp) showed no significant difference between each of the ETT groups (3.0–3.5 mm, 4.0–4.5 mm, > 5.0 mm) (Fig. 2). Differences in WOBimp between CPAP/PS and CPAP alone persisted and were the most prominent in patients with ETT 4.0–4.5 mm. We did not observe increased patient discomfort when CPAP alone was used as the Comfort B scale remained unchanged. There was no significant correlation between the time between start of MV and ERT and WOBimp. Also, there was no significant difference in WOBimp between patients who were already on CPAP/PS prior to the ERT and those on PC A/C or PC SIMV. No significant difference in WOBimp between patients with or without failed extubation was found. However, because of the low number of patients with failed extubation no firm conclusions can be made (Additional file 1, Table 2).

**Table 2:** Summary of haemodynamic and respiratory variables during extubation readiness testing using continuous positive airway pressure (CPAP) with or without added pressure support (PS)

	CPAP/PS (*n *= 110)	CPAP (*n *= 105)	Significance
PEEP (cmH_2_O)	5 (5, 5)	5 (5, 5)	0.317
Spontaneous breath rate (/min)	33 (23, 42)	37 (27, 46)	< 0.001*
SpO_2_ (%)	97 (96, 98)	97 (95, 98)	0.394
EtCO_2_ (mmHg)	5.90 (5.38, 6.65)	6.23 (5.55, 6.94)	< 0.001*
VTe (mL/kg)	7.72 (6.66, 8.97)	7.08 (5.82, 8.08)	< 0.001*
Heart rate (/min)	125 (109, 140)	125 (110, 141)	0.161
Comfort Scale	12 (10, 13)	12 (10, 13)	0.987

Data are shown as median (interquartile range)

A *p* value of < 0.05 (*) was denoted as statistically significant

### Factors independently associated with delta WOBimp

Multivariate regression analysis was used to test if ETT size, expiratory VT, Tinsp and PIFR can predict the delta WOBimp. Results showed that 15% of the variance was explained by these variables (*R*^2^ = 0.154, *F* (5, 3.499), *p *= 0.006) when corrected for the measured WOBimp during CPAP/PS ventilation. Furthermore, the size of the endotracheal tube did not contribute to ∆WOBimp (*β* − 0.030, SE 0.022, *p *= 0.171) (Table 3).

**Table 3:** Estimates of fixed effects

Dependent variable	Parameter	*β*	SE	Beta	*t*	*p* value
∆WOBimp	Baseline WOBimp	0.085	0.087	0.097	0.979	0.330
	ETT size	− 0.030	0.022	− 0.310	− 1.379	0.171
	Tinsp	0.069	0.123	0.086	0.561	0.576
	PIFR*	0.013	0.005	0.434	2.520	0.013
	VTe	0.018	0.010	0.191	0.085	0.085

The difference in imposed work of breathing between CPAP/PS and CPAP (∆WOBimp) was stated as dependent variable. The measured WOBimp during CPAP/PS was noted as baseline WOBimp. The effect of baseline WOBimp, endotracheal tube size (ETT size), inspiratory time (Tinsp), peak inspiratory flow rate (PIFR) and expired tidal volume (VTe) on ∆WOBimp was studied

A *p* value of < 0.05 (*) was denoted as statistically significant

## Discussion

This study showed that WOBimp generated during extubation readiness testing in a heterogeneous group of mechanically ventilated children with and without lung injury was significantly lower when PS was used compared to CPAP alone. However, this difference was clinically negligible because patient discomfort measured by the Comfort B did not increase when patients were tested without PS. Despite the fact that our study was not designed to test the effect of CPAP/PS versus CPAP alone on failed extubation rate, our observations may challenge the routine use of PS during extubation readiness testing, even in very young children with small ETT sizes.

It is common for paediatric critical care practitioners to use PS during extubation readiness testing [6, 7]. However, the present data questions this common practice and supports previous work by Khemani et al. who reported no clinically relevant increase in PRP as proxy for WOBphys when patients were on CPAP alone [15]. To our best of knowledge, the present study is one of the first reporting WOBimp in the paediatric context. As a consequence, there is no data on what values of WOBimp could be regarded as clinically acceptable. In adults with normal lung function, it has been reported that they need to generate approximately 0.3–0.6 J/L for expanding lungs (elastic forces, flow-resistive resistance and inertial work) and chest wall [19, 20]. Higher values can be expected when the respiratory load is increased because of increases in elastic and/or flow-resistive work. Kirton et al. reported WOBimp up to 1.1 J/L in 21 adults who were ventilated > 48 h and apparent ventilatory insufficiency observed during a weaning or pre-extubation trial [16]. They also observed that WOBimp was almost twice WOBphys and may even contribute as much as 80% to the total work of breathing, underscoring the importance of taking WOBimp into account when identifying causes underlying a failed ERT. In the present study, WOBimp values were well below or within the lower normal range of values reported in healthy adults.

The present study was not powered to detect differences in WOBimp between children who did and did not fail extubation. The extubation failure rate was 8%, which is in agreement with previously reported rates [5]. Based on the WOBimp values observed in this study, approximately 3500 patients would be needed in an observational study to establish the suitability of WOBimp as predictor for failed extubation. Also, it cannot be ruled out that the patients in the present study could have been extubated earlier. Previous data has shown that the success rate of paediatric unplanned extubation is about 50% [5]. This calls for a better implantation of daily extubation readiness testing, and that from a physiologic perspective based on the data of the present study this can be done on CPAP alone in a well-defined group of children.

Measuring WOBimp requires the insertion of a catheter in the ETT. Placement of such catheters reduces intraluminal space and will automatically result in increased flow resistance, which may be more relevant in the smallest ETT. It can be calculated that a 3.5 Fr catheter in ETT ≤ 4.5 mm resulted in a 15% reduction in intraluminal space (ETT 3.0 mm); for the larger tubes (i.e. ETT 7.5 mm) in the present study this was 5%. Reassuring, WOBimp values were the lowest in patients with ETT 3 and 3.5 mm and measured PIFRs were comparable with previously published data, indicating that the values found in this study in young children were not overestimated [8, 15, 21] (Additional file 1, Figure 1 and Figure 2). However, there is a difference in SBT duration between the two trials which possibly challenges the patients endurance. Duration of the SBT has always been a complex matter in adults and paediatrics, and no consensus has been reached yet. [22, 23]

There are a few limitations that need to be discussed. First, the present study is a single-centre study although our unit is comparable to most North-American and European centres and generalisability is high given the fact that this is a physiologic study. Second, we only included a heterogeneous group of patients extubated during office hours, thereby potentially introducing a selection bias by missing out on patients extubated during non-office hours. Third, we did not measure peak inspiratory resistance but calculated these values derived from bench testing, so the reported values of resistance may be over- or underestimated [9]. However, these limitations are not different from the ones reported in the study by Khemani et al. [15]. Fourth, the age distribution of our study population was skewed towards younger age. This limits the interpretation of the change in WOBimp between CPAP/PS and CPAP alone stratified by ETT size and calls for further study although the issue of presumed increased resistance of the ETT is only relevant for young children. Lastly, WOBimp was measured during a 5-min stable period of CPAP alone, so it cannot be ruled out that this period was too short observe signs of insufficient patient respiration. Khemani et al. used 5-min stabilisation and 5-min recording period in all patients who were at least 2 h on CPAP alone. Reassuringly, about half of the patients in the present study were already on CPAP/PS before the ERT. Their WOBimp values were not different from those who were on controlled ventilation before the ERT, so it is unlikely that the short duration of CPAP alone may have seriously affected the results in the present study.

## Conclusion

In conclusion, this study showed the WOBimp generated during extubation readiness testing in a heterogeneous group of mechanically ventilated children with and without lung injury was significantly increased when CPAP alone was used compared to CPAP/PS, although this appeared clinically irrelevant in terms of patient comfort. Our observations may challenge the routine use of PS during extubation readiness testing, even in very young children with small ETT sizes.

## Additional file

**Additional file 1.** Figures and Tables.

## Data Availability

The dataset used and analysed during this study are available from the corresponding author on reasonable request.

## Abbreviations

A/C: assist control
CPAP: continuous positive airway pressure
ERT: extubation readiness test
ET-CO_2_: end-tidal CO_2_
ETT: endotracheal tube
FiO_2_: fraction inspired oxygen
Fr: French
HR: heart rate
IRB: institutional review board
IQR: interquartile range
mPaw: mean airway pressure
MV: mechanical ventilation
NIV: non-invasive ventilation
PC: pressure controlled
PEEP: positive end-expiratory pressure
PICU: paediatric intensive care unit
PIFR: peak inspiratory flow range
PIP: peak inspiratory pressure
PRISM: paediatric RISK of mortality
PRP: pressure rate product
PS: pressure support
Ptrach: tracheal pressure
RR: respiratory rate
RSBI: rapid shallow breathing index
SBT: spontaneous breathing trial
SpO_2_: peripheral saturation
Tinsp: inspiratory time
VTe: expired tidal volume
UAO: upper airway obstruction
WOB: work of breathing
WOBimp: imposed work of breathing
WOBphys: physiologic work of breathing
WOBtot: total work of breathing
