# Supplementary material for: The effect of pressure support on imposed work of breathing during paediatric extubation readiness testing
Source: Ann Intensive Care. 2019 Jul 2;9:78. doi: 10.1186/s13613-019-0549-0 (PMC6606677; doi:10.1186/s13613-019-0549-0)
Supplement: Supplementary file 1 — Additional file 1. Figures and Tables. [file 13613_2019_549_MOESM1_ESM.docx]

**Additional file 1**

**The effect of pressure support on imposed work of breathing during paediatric extubation readiness testing**

Jefta van Dijk, MD (1) j.van.dijk01@umcg.nl

Robert G.T. Blokpoel, MD (1) r.g.t.blokpoel@umcg.nl

Alette A. Koopman, MSc (1) a.a.koopman@umcg.nl

Sandra Dijkstra, RN (1) s.k.dijkstra@umcg.nl

Johannes G.M. Burgerhof, MSc (2) j.g.m.burgerhof@umcg.nl

Martin C.J. Kneyber, MD PhD FCCM (1, 3) m.c.j.kneyber@umcg.nl

(1) Department of Paediatrics, division of Paediatric Critical Care Medicine, Beatrix Children’s Hospital, University Medical Center Groningen, University of Groningen, Groningen, the Netherlands;(2) Department of Epidemiology, University Medical Center Groningen, The University of Groningen, Groningen; (3) Critical care, Anaesthesiology, Peri-operative & Emergency medicine (CAPE), University of Groningen, Groningen, the Netherlands

Effect of PS on peak inspiratory flow rates and resistance

Figure 1 graphically depicts the PIFR and calculated peak inspiratory resistance as function of ETT size and PIFR. Distribution of the ETT sizes in the cohort is summarized in Table 1 of the electronic supplemental material (ESM). Sixty-eight percent of the studied patients had an ETT ≤ 4.0 mm. Calculation of the peak inspiratory resistance was limited to ETT sizes ≤ 6.0 mm, resulting in exclusion of 4 measurements (ETT 6.5 mm – 7.5 mm). During CPAP/PS, PIFR was the lowest in patients with the smallest ETT (4.6 (IQR 4.4 – 7.0) L/min) and increased with increasing ETT size ( 19.7 (IQR 17.7 – 33.0) L/min). The peak inspiratory resistance was the largest in patients with the smallest ETT (37.2 (IQR 36.2 – 46.3) cmH_2_O/L/sec) and decreased with increasing ETT size (7.2 (IQR 6.0– 12.5) cmH_2_O/L/sec). Comparable results were found when patients were on CPAP without PS for both PIFR, with 4.7 (IQR 3.8 – 6.3) L/min with the smallest ETT to max 15.7 (IQR 14.6 – 31.9) L/min. The peak inspiratory resistance decreased from 37.6 (IQR 34.0 – 43.6) cmH_2_O/L/sec in a 3.0 mm ETT to 5.5 (IQR 5.1 – 12.0) cmH_2_O/L/sec in a 6.0mm ETT. However, all of these values were within the expected range.

**Table 1**

Ventilator characteristics of the studied cohort. Extubation failure is defined as reintubation or use of non-invasive ventilation within 48 hours after extubation. Three patients failed extubation due to upper airway obstruction and were excluded from further analyses.

|  |  | **N (%)**  **N=112** |
| --- | --- | --- |
| Ventilator mode | Pressure A/C | 58 (51.8%) |
|  | CPAP/PS | 54 (48.2%) |
| Tube position | Oral | 54 (48.2%) |
|  | Nasal | 58 (51.8%) |
| Tube size | 3.0mm | 9 (8.0%) |
|  | 3.5mm | 35 (31.2%) |
|  | 4.0mm | 33 (29.4%) |
|  | 4.5mm | 17 (15.2%) |
|  | 5.0mm | 5 (4.5%) |
|  | 5.5mm | 6 (5.4%) |
|  | 6.0mm | 3 (2.7%) |
|  | 7.0mm | 3 (2.7%) |
|  | 7.5mm | 1 (0.9%) |
| Extubation outcome | Succes | 103 (92.0%) |
|  | Failure | 9 (8.0%) |

**Table 2**

Outcome measures following extubation readiness testing using continuous positive airway pressure (CPAP) with or without added pressure support (PS). Nine patients required re-intubation or non-invasive ventilation within 48 hours and were labeled as failed extubation. Data are shown as median (interquartile range). The *p*-value shown in the table is a result of a Mann-Whitney U analysis between patients who failed or succeed extubation. A Wilcoxon rank test was performed to analyze differences between the two paired study moments (^1^ *p* < 0.001; ^2^ *p* < 0.05).

|  | **Extubation outcome** | **CPAP/PS**  **(n=110)** | ***p-value*** | **CPAP**  **(n=105)** | ***p-value*** |
| --- | --- | --- | --- | --- | --- |
| **WOBimp**  **(Joules/L)** | **Succes** | 0 (0, 0.1)^1^ | 0.73 | 0.26 (0.19, 0.49)^1^ | 0.14 |
|  | **Failure** | 0.00 (0, 0.26)^2^ |  | 0.31 (0.26, 0.65)^2^ |  |
| **PIFR**  **(L/min)** | **Succes** | 9.0 (6.4, 16.7)^1^ | 0.85 | 8.4 (5.8, 11.6)^1^ | 0.72 |
|  | **Failure** | 9.7 (6.7, 15.3) |  | 9.0 (5.5, 10.4) |  |
| **Rpiek**  **(cmH_2_O/L/min)** | **Succes** | 24.0 (17.3, 28.4)^1^ | 0.11 | 20.3 (15.5, 26.1)^1^ | 0.15 |
|  | **Failure** | 27.1 (21.4, 37.8)^2^ |  | 23.4 (17.4, 35.6)^2^ |  |
| **RSBI**  **(f/Vt)** | **Succes** | 3.8 (2.6, 5.7)^1^ | 0.04 | 4.9 (3.3, 6.7)^1^ | 0.02 |
|  | **Failure** | 6.1 (4.1, 7.1)^2^ |  | 7.7 (4.9, 8.5)^2^ |  |
| **Comfort Score** | **Succes** | 12 (10, 13) | 0.72 | 12 (10, 13) | 0.61 |
|  | **Failure** | 12 (10, 14) |  | 1. (10, 14) |  |

**Figure 1**

Peak inspiratory resistance calculated as a function of measured peak inspiratory flow rates (L/min) for different endotracheal tube sizes. Peak inspiratory flow rate was read from the ventilator; peak inspiratory resistance was calculated using the bench data and fitted equations by Manczur et al [1] and Khemani et al [2]. Outliers were identified using the Grubbs test (Alpha = 0.05) and subsequently removed from calculations.

**
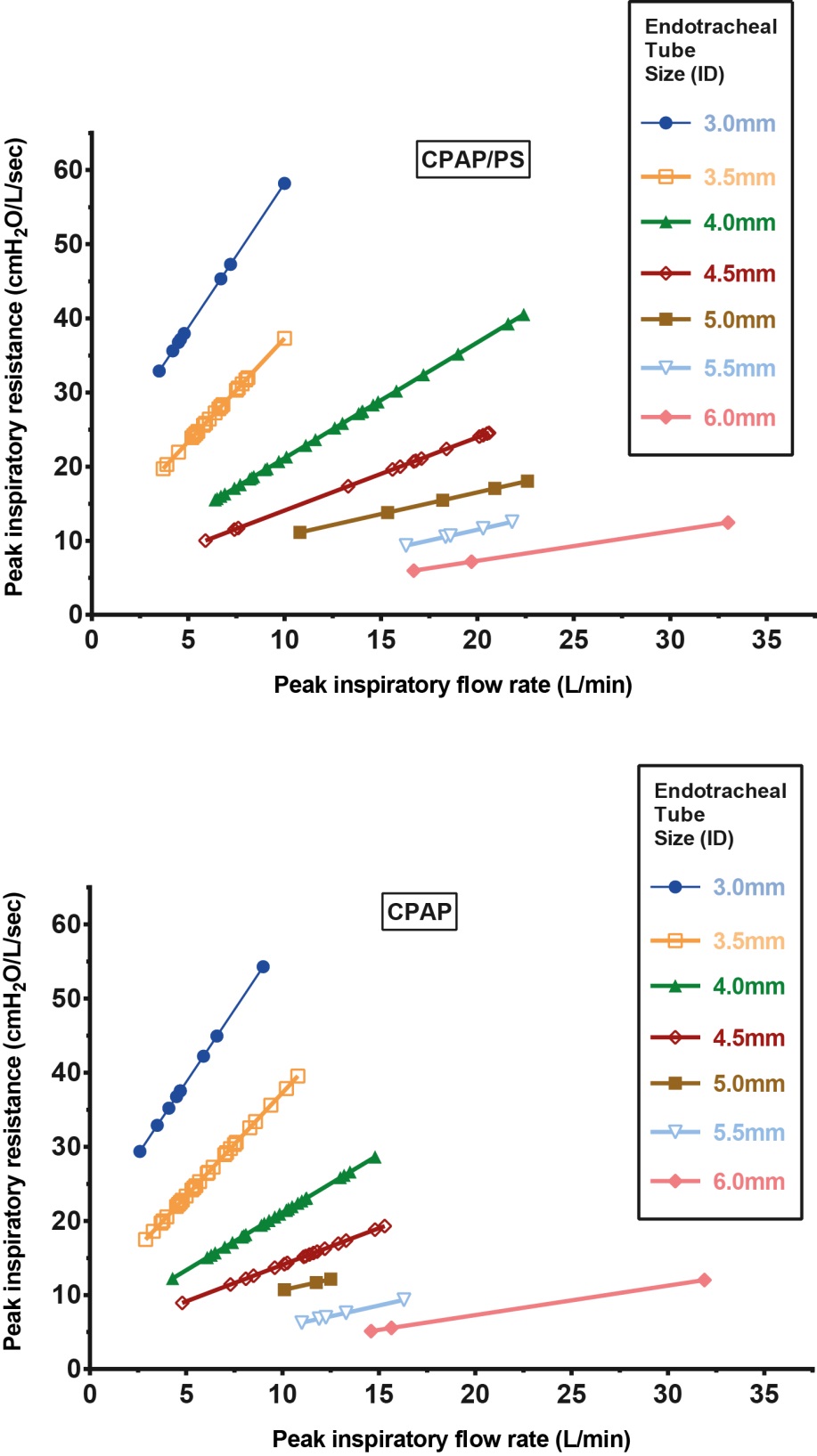
**

**Figure 2**

Ranges (displayed as shaded areas representing minimum to maximum) of peak resistance (cmH_2_O/L/s) calculated using peak inspiratory flow rate (L/min) measuring during extubation readiness testing without added pressure support, superimposed on bench data of endotracheal tube resistance from Manczur et al [1] and Khemani et al [2].


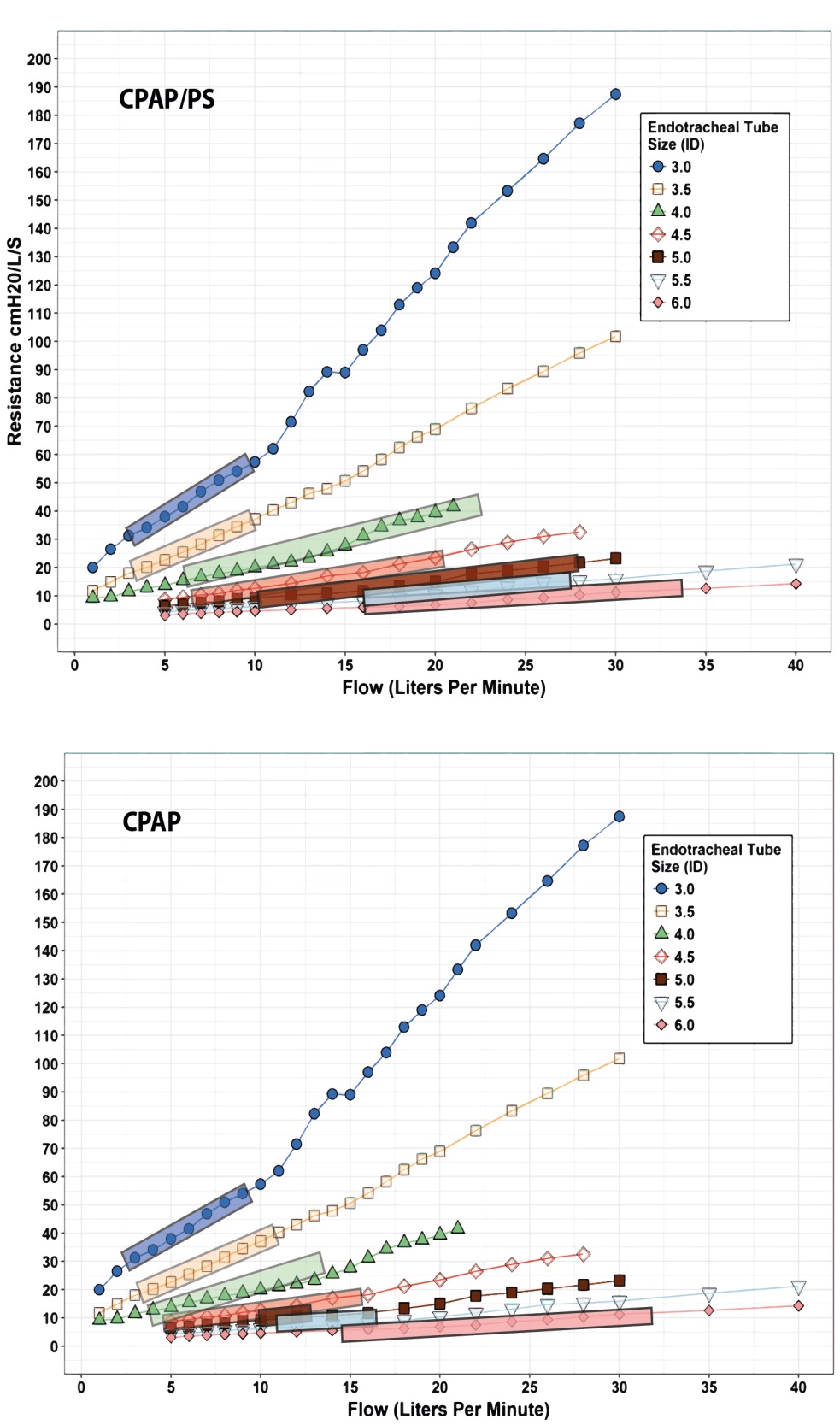


**References**

1. Manczur, T, Greenough, A, Nicholson, GP, Rafferty, GF. Resistance of pediatric and neonatal endotracheal tubes: influence of flow rate, size, and shape. Crit Care Med. 2000;28(5):1595-8.

2. Khemani, RG, Hotz, J, Morzov, R, Flink, RC, Kamerkar, A, LaFortune, M*, et al* Pediatric extubation readiness tests should not use pressure support. Intensive Care Med. 2016;42(8):1214-22
